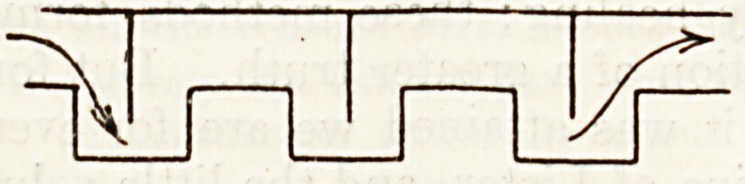# A Carefully Thought-Out System

**Published:** 1921-03-26

**Authors:** 


### DUTCH ANTI-TUBERCULOSIS WORK.

### A Carefully Thought-out System.

In Amsterdam the principal tuberculosis dispensary occupies the whole of a former private house, situated picturesquely on the banks of the Keizersgracht, one of the numerous tree-fringed canals threading this capital city. The transformation to anti-tuberculosis purposes has been effected with true Dutch neatness and order. In Holland generally, and particularly in Amsterdam, houses are high and narrow, by reason of being built upon piles sunk in the alluvium. All five floors of the building have been made good use of, as also part of the adjoining premises. Beginning below, there is accommodation for a small clerical staff, and behind it a first waiting-room. Up a few steps comes a second waiting-room giving access to the consulting-room. This has three separate dressing apartments, each opening either way; and as soon as one is vacated the fact is automatically signalled near the physician's table. On the second floor is a room devoted to the records; there is a card-index system, and two case-sheets for each case, one for the patient, the other for the patient's family. An assistant-physician's consulting-room is also found here. Higher again is an $x$-ray installation, the "dark room" attached to which deserves attention: light is excluded (and satisfactorily so), not by a door, with its risks of sudden opening, but by a zigzag entrance always patent. A former bathroom, tiled throughout, serves admirably for a laboratory, and the medical director's room is above all. Here were found several German and Austrian tuberculosis journals, the well-known international double-cross *Tuberculosis* in Dutch, the two English tuberculosis journals, and the American one. Throughout the building—at least, those parts of it frequented by patients—are hung the lively popular posters which the American Red Cross caused to be printed in France recently: their letter-press must be unintelligible to many of their audience, but the pictures make a universal appeal.

### Objects and Maintenance.

Although on occasion beds are given out to patients, as also a neat kind of washing-tub to help in the separate washing of his clothes, the object of the work here is wholly diagnostic: no treatment is attempted, not even the administration of cod-liver oil. This is perhaps a pity, especially as war experience has shown that the origin of tuberculosis is not so much a matter of infection as of depressed constitutional resistance. The direction of the undertaking is private, but the municipality contributes largely to its maintenance. Perhaps a little of the work undertaken is supererogatory; for instance, our representative saw in a nurse's (or "sister's," as the Dutch say) report on a patient's home conditions, an admirably drawn plan of the sick man's house. It certainly rounded off very neatly the *dossier* of the case, but perhaps her time would have been better spent in helping, say, with artificial pneumothorax treatment. Any serious case is sent to hospital, and the dispensary physicians have the right to admit cases there direct. These general hospitals are maintained by the municipality. Not so the sanatoriums, which are private; here the dispensary pays for each case sent in. Two other dispensaries, in other parts of the city, are in connection with the principal one. The sanatoriums are situated in various parts of Holland, several of them near Amsterdam. There are also two marine institutions ("Zee-Hospitium").

### Zeehospitium Katwyk-aan-Zee.

This institution, founded in 1906 and enlarged in 1908 and 1914, is situated amongst the sand-dunes which are Holland's ramparts against marine irruptions. Founded by private charity, it is largely supported by the Rotterdam municipality, but also takes patients from all parts of Holland, notably, as already mentioned, Amsterdam. It accommodates some 180 children. These are by no means always cases of bone and joint tuberculosis, but also scrofula, pyogenic scrofulosis, latent juvenile tuberculosis, and incipient pulmonary patients. There are two resident doctors, a "directrice" of nurses, and sisters. The elder children are housed in a separate building, and there is an isolation hospital.

There is an $x$-ray installation, a "Verbands-kammer," or plaster-room, and a German artificial "Hohensonne" lamp; there is also a very commodious theatre, the seaward wall of which is one huge sheet of glass, through which appears a scene like a picture by Ruysdael. Electricity is generated on the premises, and there are sea-water baths, hot and cold. Glass-roofed "Liege-hallen" (the climate is rather boisterous) provide change of scene for the little patients, and schooling is available, either outdoor in a hollow of the dunes, or else indoor in airy schoolrooms. A hand trolley running on rails furnishes easy transport. The senior medical resident has studied at Berck-sur-mer, which, with Ruppertshain, is the model of the Treloar Hospital at Alton, and, indeed, of all up-to-date institutions for surgical tubercle; but it would perhaps be an advantage if the bone and joint cases, which require heliotherapy and, indeed, a much more advanced therapeutic technique than do the remainder, were accommodated exclusively here; scrofula and malnutrition could be treated as well at juvenile convalescent homes, outdoor schools, etc.

For a country of but six millions, albeit with rather large tropical colonies, the above anti-tuberculosis measures are far from being behindhand. No doubt in Holland, as elsewhere, the pressure of public opinion as to social amelioration will soon augment them considerably.

## Figures and Tables

**Figure f1:**